# Response to “How much does the Addiction-Like eating behaviour scale add to the debate regarding food versus eating addictions?”

**DOI:** 10.1038/ijo.2017.291

**Published:** 2018-05-15

**Authors:** H K Ruddock, P Christiansen, J C G Halford, C A Hardman

**Affiliations:** 1 School of Psychology, University of Birmingham, Birmingham, UK; 2 UK Centre for Tobacco and Alcohol Studies, UK

We thank Schulte, Potenza, and Gearhardt for their response to our recent publication (The development and validation of the Addiction-like Eating Behaviour Scale;AEBS). The AEBS quantifies individual differences in core behavioural processes that characterize overeating, and which are similar to the processes underpinning drug/alcohol use and other compulsive behaviours. This is distinct from the Yale Food Addiction Scale (YFAS) which is based on the DSM substance-use disorder criteria ^1, 2^. However, as Schulte *et al.* point out, both the AEBS and YFAS incorporate behavioural criteria and this is consistent with the general assessment of addictive disorders (substance-based *and* behavioural).

With regard to the distinction between substance-based and behavioural addictions, Schulte *et al.* suggest that the AEBS is consistent with a substance-based framework due to the inclusion of items referring to problematic intake of ‘high fat/sugar’ foods. We contest this view and point out that these items refer to general *types* of food, rather than a specific ingredient (as a substance-based framework would predict). This is consistent with evidence that people experience problems controlling their intake of a *range* of energy-dense foods ^3, 4^. This implies that there is not a specific addictive ingredient in foods but rather it is the high-energy density of such foods which makes them highly desired. Notably, a recent study found that YFAS symptoms were more closely related to the overconsumption of foods high in fat *and* sugar (i.e. energy-dense foods), than to foods high in sugar alone ^5^.

Schulte *et al.* also suggest that a move away from the DSM criteria for addictive disorders limits the validity of the AEBS as a measure of addiction. However, given fundamental differences between drugs and food ^4, 6^, we suggest that moving away from the DSM criteria *is* necessary to develop a valid framework for assessing addiction-like eating. Our approach led to the development of a scale which is entirely consistent with theoretical perspectives on addiction. Specifically, the two-factor structure of the AEBS (appetitive drive/dietary control) reflects well-established dual-process models of addictive disorders *and* overeating ^7, 8^ (i.e. increased reward responsivity/diminished inhibitory control). Furthermore, individual scale items of the AEBS correspond with core features of addictive disorders (e.g. loss of control, preoccupation, negative consequences)^9^. It is also important to note that the AEBS provides a *continuous* measure of individual differences in addition-like eating, and was *not* intended as a diagnostic tool for ‘eating-addiction’.

Finally, Schulte *et al.* suggest that the behavioural eating addiction vs. food addiction debate detracts from key issues surrounding the concept of addiction-like eating. However, we suggest that such issues can only be addressed following careful consideration of how addiction-like eating should be defined. The AEBS provides a means to assess addiction-like eating behaviour in a way that reflects validated models of motivated behaviour. We agree with Schulte *et al.* that establishing the distinction between food addiction and binge eating is a key area for future research ^10^. The AEBS may help to address this; indeed, the scale was able to predict variance in BMI *beyond* that accounted for by a measure of binge eating. We therefore envisage that the AEBS will have important implications for establishing the clinical utility of addiction-like eating, and enabling the development of personalised treatments for overeating and obesity.
